# Treatment for Recurrent Ovarian Cancer—At First Relapse

**DOI:** 10.1155/2010/497429

**Published:** 2009-12-24

**Authors:** Kimio Ushijima

**Affiliations:** Department of OBGYN, Kurume University School of Medicine, 67 Asahi machi, Kurume 830-0011, Japan

## Abstract

Recurrent ovarian cancer is a lethal disease, and few patients can be cured. Although most patients receive standardized surgery and chemotherapy, the status of recurrent disease is heterogeneous. The site of recurrence and the survival intervals after recurrence are also widely distributed. Among a number of factors, many clinical trials identified time to recurrence was the factor most related to chemosensitivity at first relapse. The current recommendation for platinum sensitive ovarian cancer is a carboplatin containing combination chemotherapy. Generally, a single agent is chosen for platinum resistant ovarian cancer. Patients with single site recurrence and a long disease free interval are candidates for secondary cytoreduction, which may provide longer survival. There are several treatment choices at first relapse, and disease status, chemotherapy-free interval, and the patient's condition play a major role in the decision making process.

## 1. Introduction

Ovarian cancer has the second highest incidence of any gynecologic malignancy in western countries. In Asian countries, ovarian cancer has the third highest incidence, but it is rapidly increasing. In spite of recent progress in treatment strategy, it is still the leading cause of death among cases of gynecologic cancer. After recurrence, generally 70% of advanced stage ovarian cancer relapses, and even in stage I or II patients, the relapse rate is 20%–25%. The survival curve after recurrence never plateaus, which means that the goal of treatment for recurrent ovarian cancer is controlling the disease and disease-related symptoms, limiting treatment-related toxicity, and maintaining or improving quality of life [1]. Nevertheless, the period up to first relapse varies widely, from a few months to more than 5 years. Several prognostic factors have been reported, and clinical trials have provided us with some treatment options. In this paper, patterns and treatment for ovarian cancer at first relapse are discussed.

## 2. Pattern and Classification of Recurrence

The median interval to first recurrence is 18 to 24 months in ovarian cancer. To clarify the prognostic factors and to determine the treatment procedure, grouping of recurrent patients was applied. They were distinguished by pretreatment or initial treatment profiles, such as FIGO stage, histologic type, and size of residuals. Furthermore, the status at recurrence, such as time to recurrence, site of recurrence, number of recurrent sites, and treatment procedure was also tested as prognostic factors. Time to recurrence was divided to three groups as follows: more than 12 months, less than 6 months, and 6 to 12 months. Sites of first recurrence were divided to two groups, primary site (pelvis and abdominal cavity), and other areas. The number of recurrent sites was divided into two groups: single and multiple. Treatment procedure was divided into surgery and surgery plus chemotherapy.

Half of the recurrences occur at more than 12 months from the end of the first-line therapy, and one quarter of all recurrences occur at less than 6 months. Regarding recurrent sites, Table 1 shows the distribution of first relapse sites from our data on 112 recurrent cases. Fifty-five percent of first relapse was found at the primary site (pelvis or abdomen); the rest was found at distant lesions similar to previous reports [16]. There was a wide variety of recurrent sites, such as, retroperitoneal nodes, liver or spleen, brain, and bone. There was no difference in first recurrent sites between early and advanced stage cancers [17]. 

An Italian study showed statistical significance between survival from recurrence and initial clinical stage (I, IIA versus IIB–IV), residual disease after initial surgery (≤1 cm versus >1 cm), time to recurrence (≤6 months versus 6–12 months, >12 months), and treatment at recurrence (surgery plus chemotherapy versus others) by univariate analysis. In multivariate analysis, residual disease and time to recurrence were the only two independent prognostic factors after recurrence. Conversely, histological type (serous versus non serous), tumor grade (G1 versus G2, G3), number of recurrence sites (single versus multiple), and symptoms at recurrence (symptomatic versus asymptomatic) had no prognostic relevance [16]. Hawkins identified predictive factors of survival after first relapse by time to progression (TTP) (>593 days), original tumor grade ≤2, and performance status ≤2 using tree model analysis. In this analysis, the good response group showed longer survival than the intermediate or poor response groups [18]. Data from our 110 recurrence cases showed that time to recurrence (≤6 months versus >6 months), the number of recurrence sites (single versus multiple), and treatment at recurrence (chemotherapy plus surgery and/or radiotherapy versus chemotherapy only) had statistical significance in survival after recurrence. On the other hand, histologic type (serous or endometrioid versus mucinous or clear cell) and initial clinical stage (I, II versus III, IV) did not have any correlation with the survival interval after recurrence (Ushijima, unpublished data).

## 3. Treatment Option for Recurrence

### 3.1. Chemotherapy

#### 3.1.1. Patient Selection

**Figure 1:**
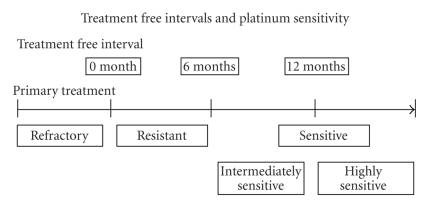
Treatment free intervals and platinum sensitivity.

Regarding the secondary response of platinum-based chemotherapy, Markman clarified patients with more than 24-month platinum free interval as showing superior response compared to patients with between 5- and 12-month interval (59% and 27%, resp.) [22]. Gore reported that patients with at least an 18-month progression free interval (PFI) showed a remarkably higher response rate compared to patients with less than 18 months of PFI (53% versus 17%) [23]. This theory was supported by the following literature. It is generally accepted that response to second-line chemotherapy correlates well with PFI, from the end of initial treatment [24]. For the selection of a chemotherapeutic regimen at first relapse, patients are categorized according to their estimated chemosensitivity depending on PFI. Many studies have employed the 6-month interval as the borderline when defining the criteria [25]. Figure 1 explains the criteria of platinum sensitivity. Patients whose disease showed initial response to platinum and recurred having >6 months PFI are defined as platinum sensitive. Patients whose disease showed response to or stable disease prior to platinum treatment and who recurred within 6 months of final treatment are defined as platinum resistant. For a more strict separation of the platinum sensitive disease, patients with a more than 12-month interval are defined as highly sensitive disease and patients with 6 to 12 months are considered intermediate sensitive disease [26]. Patients, whose disease progressed during platinum treatment and have less than 3-month platinum free interval, are defined as refractory disease, which means that these patients have very little chance to respond to a platinum-based therapy [27].

#### 3.1.2. Chemotherapy for Patients with Platinum Sensitive Ovarian Cancer

The single use of carboplatin has been a standard regimen for the patients with platinum sensitive disease. A phase II trial on paclitaxel and carboplatin combination for sensitive (≥6months) patients showed a considerably high response rate (90%) and 9 months PFI among 20 measurable and assessable patients [2] (Table 2). The ICON4/AGO-Ovarian Cancer 2.2 trial which was a large international phase III study showed that paclitaxel and platinum combination had a statistically higher response rate and improved progression free survival (PFS) than conventional platinum combination in 802 patients with platinum sensitive disease [6]. Nevertheless, this trial had only 34% of the patients who were treated with paclitaxel in the front-line chemotherapy and included several combinations in the treatment regimens as conventional platinum-based chemotherapy.

A Spanish trial was conducted by more stringent design. Eighty-one patients with platinum sensitive disease were randomized to receive carboplatin (AUC: 5) alone as a standard arm, or paclitaxel (175 mg/m^2^) and carboplatin (AUC: 5) as an experimental arm. More than 80% of patients received paclitaxel previously. Those who were treated with paclitaxel and carboplatin had a significantly higher response rate (75.6% versus 50.0%) and a PFS that was 4 months longer than those treated with carboplatin only [7]. Furthermore, the platinum and paclitaxel combination did not increase toxicity except moderate neurotoxicity. These results suggested the benefits of a paclitaxel and platinum combination and it became the standard treatment of choice for the patients with platinum sensitive disease (Table 3).

A phase I, II study of gemcitabine and carboplatin combination showed 62.5% of objective response in patients with platinum sensitive ovarian cancer [3] (Table 2). According to this result, a large randomized study was conducted by AGO, NCIC CTG, EORTC intergroup trial to compare gemcitabine (1.000 mg/m^2^) plus carboplatin (AUC: 4) with carboplatin (AUC: 5) alone for platinum sensitive ovarian cancer. In total, 356 patients were recruited and higher response rates were shown in the gemcitabine plus carboplatin combination including a higher CR rate (14.6% versus 6.2%) and a significantly longer PFS (8.6 months versus 5.8 months). There was no difference in nonhematologic toxicities, but grades 3 and 4 hematologic toxicities were greater with the combination [8] (Table 2).

Pegylated liposomal doxorubicin (PLD) is one of the alternatives for platinum resistant ovarian cancer. A phase II study of PLD (30 mg/m^2^) plus carboplatin (AUC: 5) q/4weeks combination chemotherapy showed a 46% objective response rate for the patients who recur within six to twelve months [4]. A randomized phase III study of PLD (30 mg/m^2^) plus carboplatin (AUC: 5) q/4w versus carboplatin (AUC: 5) q/4w alone for platinum sensitive ovarian cancer was done by SWOG. The PLD containing regimen showed a 4-month longer PFS (12 months versus 8 months). The lack of incidence of carboplatin-related allergic reaction in the PLD-treated patients may be an additional benefit of this combination [9]. Gynecologic Cancer Intergroup (GCIG) conducted a large phase III study for patients with taxane pretreated sensitive relapse disease, which consisted of PLD (30 mg/m^2^) plus carboplatin (AUC: 5) q/4weeks (CD) versus retreatment by paclitaxel (175 mg/m^2^) plus carboplatin (AUC: 5) q/3weeks (CP) (CALYPSO study), and the result was presented at ASCO in 2009. The number of patients was 466 in CD and 508 in CP. When comparing median PFS, CD showed statistically longer PFS (11.3 months versus 9.4 months). These two regimens had different toxicity profiles. CD had more incidences of thrombocytopenia and palmar-plantar erythrodysesthesia (PPE). Nevertheless, CD had extremely less incidence of Grade 4 neutropenia, Grade 2 alopecia, Grade 2–4 neurotoxicity, and carboplatin hypersensitivity reaction, which resulted in significantly less incidence of discontinuation of treatment by toxicity than CP. This combination may be a good option for platinum or taxane sensitive relapse [10].

Docetaxel showed similar response to paclitaxel and a different toxicity profile in first line chemotherapy [28]. The Japanese group conducted a phase II trial with docetaxel (70 mg/m^2^) and carboplatin (AUC: 5) combination for platinum sensitive patients. The objective response rate was 59% (17/29, including 5 CR) in 29 evaluable patients (Table 2). They showed 46% sensory neurotoxicity and only 7% motor neurotoxicity, without any grade 3 or 4 neurotoxicity, even though most patients were previously treated by paclitaxel [5]. According to this result, a new trial which consisted of biweekly docetaxel (35 mg/m^2^) with bolus carboplatin (AUC: 5) repeated every 4 weeks is now ongoing. We can expect a similar response to bolus DC treatment with less hematologic toxicity.

In summary, a carboplatin-based combination is strongly recommended for patients with platinum sensitive disease rather than carboplatin monotherapy. Paclitaxel with carboplatin is the most frequently used combination and showed favorable result for these patients. Nevertheless, alternative combinations of gemcitabine or PLD with carboplatin have responses and prolonged survival rates similar to paclitaxel and carboplatin with different toxicity profiles. Gemcitabine combination showed similar bone marrow toxicity but less neuropathy or alopecia. PLD combination showed less neurotoxicity or bone marrow toxicity, but more PPE.

#### 3.1.3. Chemotherapy for Patients with Platinum Resistant Ovarian Cancer

Many phase II trials of single agents for patients with platinum resistant disease showed at most only a 5%–20% response rate (Table 4). Therefore, duration of disease control and low incidence of toxicity should be an important factor in choosing the proper drugs [29]. PLD demonstrated a response in the treatment of recurrent ovarian cancer in some phase II studies [30, 31]. Although the recommended dose of PLD is 50 mg/m^2^ q/4weeks, a reduced dose (40 mg/m^2^ q/4weeks) showed a lower incidence of PPE which is schedule limiting toxicity. So, a modified dose 40 mg/m^2^ q/4weeks may be used for patients with platinum resistant ovarian cancer to minimize adverse effects [32]. PLD is recognized as the first choice nonplatinum agent for patients with relapse, who have failed first-line therapy, or who cannot tolerate platinum retreatment due to toxicity [33].

Gemcitabine has less toxicity except for manageable neutropenia. Single use gemcitabine (1000 mg/m^2^ on day 1, 8, and 15 q/4w) is well tolerated and showed 16% of partial response in 51 platinum-paclitaxel refractory ovarian cancer (Table 4) [11]. Dose limiting toxicity is bone marrow suppression; so starting at reduced doses (800 mg/m^2^) for heavily pretreated patients is reasonable [11]. A randomized phase III study comparing gemcitabine (1000 mg/m^2^ on day 1, 8 q/3w) with PLD (50 mg/m^2^ q/4w) in platinum resistant patients showed similar response and PFS. Fatigue (grade2) is frequently the worst toxicity, nausea (grade3) and neutropenia (grade3 and 4) are also statistically frequent in gemcitabine, and PPE is more frequent in PLD [14] (Table 4). The different mechanisms and noncross-resistance of gemcitabine can be expected to overcome drug resistance in combination with other nonplatinum drugs [34]. A combination of gemcitabine (1000 mg/m^2^) with weekly paclitaxel (80 mg/m^2^) on days 1, 8 and 15 q/4w showed a 40% response rate and the median PFS was 5.7 months for 35 patients with platinum resistant disease [12]. A large multicenter phase II study of combination of PLD (30 mg/m^2^) day 1 and gemcitabine (1000 mg/m^2^) days 1 and 8 every 3 weeks showed a 22% overall response and a 32% stable disease for patients with platinum resistant disease. In that study, the lower PLD dose might contribute to the very low incidence of PPE (Table 5) [19].

Topoisomerase inhibitors, topotecan, irinotecan, and oral etoposide can also be used for platinum resistant disease. The standard treatment of topotecan is 1.5 mg/m^2^ for five consecutive days, every 3 weeks. The response rate for platinum resistant ovarian cancer was 12%–14% [15, 35]. High bone marrow toxicity was seen (82% of patients with Grade 4 neutropenia) in heavily pretreated patients [19]. Recently, a lower dose (1.0 mg/m^2^) 5-day q/3w protocol [36], weekly schedule (2.5 mg/m^2^ days 1, 8, 15 q/4w) [13], or 3-day schedule (1.5 mg/m^2^ days 1–3 q/3w) [37] improved treatment tolerability for heavily pretreated patients (Table 4). The combination of lower dose of PLD (30 mg/m^2^) and topotecan (1 mg/m^2^ for days 1–5) was tested for platinum resistant ovarian cancer. Although relatively higher response rate (28%) was seen in a phase II study, severe bone marrow toxicity (grade 3/4 thrombocytopenia in 41% of cases) limited further clinical use (Table 5) [20].

A randomized phase III study for topotecan and PLD for refractory or recurrent ovarian cancer was conducted. The PFS rates were similar between two arms. In patients with platinum sensitive disease, PLD was demonstrated to be significantly superior to topotecan in overall survival. On the other hand, in the platinum-refractory subgroup there was no statistically significant survival trend in favor of either liposomal doxorubicin or topotecan (Table 4) [38].

## 4. Surgery

Complete response by chemotherapy for recurrent ovarian cancer is rare, and shrinkage of the tumor does not always ensure prolongation of survival. A surgical approach may bring clinical benefit to some patients. Surgery for clinical recurrence is defined as secondary cytoreductive surgery, similar to surgery for persistent disease at the completion of chemotherapy. In multivariate analysis, disease free interval, the number of sites of recurrence, and residual disease after secondary cytoreduction were factors found to influence prognosis. The patients with the longer disease free interval (>30 months) and a single site of recurrence were most likely to reap the benefits of secondary cytoreduction (Table 6) [21]. A longer period of PFS and complete resection at secondary cytoreductive surgery are common favorable prognostic factors [39–41]. Nevertheless, the surgical result is dependent on the number of sites and the skill of the surgeon. Onda et al. proposed four prognostic factors as follows: >12 months PFS, no liver metastasis, solitary tumor, and <6 cm tumor size. Patients with three or all four of these factors who received complete surgical resection at secondary cytoreduction showed a favorable prognosis [42]. 

At secondary reduction, bowel or other organ resections are often also performed. More than 30% of surgeries included bowel resection [21], and some of them accompanied considerable morbidity, such as colostomy or pelvic exenteration [40]. On the other hand, patients with longer PFS are also expected high response to second-line chemotherapy. Therefore, careful consideration must be made when deciding which strategy, surgery, or chemotherapy to use to most benefit each patient.

## 5. Conclusion

Recurrence of ovarian cancer is a lethal and chronic disease. Nevertheless, patients with recurrent platinum-sensitive ovarian cancer may have increased response rates and longer PFS when treated with combination platinum-based chemotherapy compared to carboplatin alone. Most recurrent patients with platinum resistant disease have little chance for a long PFS, but less toxic treatment may contribute to extending their survival interval. Complete secondary cytoreduction combined with further adjuvant therapy at the time of relapse may improve clinical outcome in selected patients. There are several treatment choices from first relapse to terminal state; however these choices cannot be made uniformly. They should be decided on an individual basis depending directly on the patients' condition.

## Figures and Tables

**Table 1:** Site distribution at first relapse of ovarian cancer (*n* = 112).

Abdominal cavity	33 (29.4%)*
Pelvic cavity	29 (25.9%)*
Vaginal stump	17 (15.2%)
Retroperitoneal lymph node	8 (7.1%)
Superficial lymph node	7 (6.3%)
Liver, spleen	7 (6.3%)
Bladder	3 (2.7%)
Bone	3 (2.7%)
Brain	2 (1.8%)
Lung	2 (1.8%)
Adrenal	1 (0.9%)

Select one main site in case of multilocated.

*Recurrence at the primary site.

Kurume University 1990–2005.

**Table 2:** Phase II study results for platinum sensitive recurrent ovarian cancer.

	PTX/CBDCA	GEM/CBDCA	PLD/CBDCA	DTX/CBDCA
Author/group	Rose et al. [2]	du Bois et al. [3]	Power et al. [4]*	Ushijima et al. [5]
		AGO		WJGOG

Dose (mg/m^2^)	135/AUC 5-6	1000/AUC 4	30/AUC 5	70/AUC 5
Number of patients	20	25(16)**	54	29
Response rate (%)	91	63	46	59
PFS (months)	9	10	10	11
OS (months)	10	18	19.1	NA

PTX: paclitaxel; CBOCA: carboplatin; GEM: gemcitabine.

PLD: pegylated liposomal doxorubicin.

PFS: progression free interval; PS: overall survival.

*Patients who recur within 6 to 12 months.

**Patients with measurable disease.

NA: not applicable.

**Table 3:** Phase III study results for platinum sensitive recurrent ovarian cancer.

	PTX/CBOCA (COOP)	PTX/CBOCA	GEM/CBOCA		PLO/CBOCA	PLO/CBOCA
	versus Pt combination	versus CBDCA	versus CBDCA		versus CBDCA	versus PTX/CBOCA
Dose (mg/m^2^)	175–185/ AUC 5 (50–75)	175/AUC 5	1000/AUC 4		30/AUC 5	30/AUC 5
	versus AUC 5 (50–75)	versus AUC 5	versus AUC 5		versus AUC 5	versus 175/AUC 5
Author		González-Martín et al.	Pfisterer et al.		Alberts et al.	Pujiade-Lauraine et al.
	ICON4/ [6]	GEICO [7]	AGO OVAR, [8]		SWOG [9]	GClG [10]
Study group	AGO-OVAR2.2		NCIC CTG, EORTC GCG			
Number of patients	392 versus 410	41 versus 40	178 versus 178		31 versus 30	466 versus 508
Response rate (%)	66 versus 54	75.6 versus 50.0*	47.2 versus 30.9*		52 versus 29	NA
PFS (months)	12 versus 9*	12.2 versus 8.4*	8.6 versus 5.8*		12 versus 8*	11.3 versus 9.4*
OS (months)	29 versus 24*	NA	18 versus 17.3		26 versus 18*	NA

PTX: paclitaxel; PT: cisplatin or carboplatin.

CBDCA: carboplatin; GEM: gemcitabine; PLD: pegylated Iiposomal doxorubicin.

PFS: progression free interval; PS: overall survival.

NA: not applicable.

*Statistically significant.

**Table 4:** Study result for platinum resistant recurrent ovarian cancer (single agent).

	PLD	GEM	Topotecan	PLD versus	PLO versus GEM
				Topotecan	
Dose	50 mg/m^2^/4w	1 g/m^2^	1.5 mg/m^2^ d1–5/3w	50 mg/m^2^/4w	50 mg/m^2^/4w

		d1.8.15/4w	(1 mg/m^2^/3w)	1.5 mg/m^2^ for 5 d/3w	19/d1.8/3w
					
Author	Gordon et al. [31]	Markman [11]	Bookman et al. [15]	O'Malley et al. [13]	Mutch et al. [14]
			(Rodriguez et al. [36])		
					
Number of patients	82	51	112 (37)	130 versus 124	96 versus 99
Response rate					
CR + PR (%)	18.3	16	12.4 (22 )	12.3 versus 6.5	8.3 versus 6.1
+ SD (%)	66.1	NA	NA(44)	40 versus 49.2	46.9 versus 60.6
					
Most frequent					
Adverse effect	PPE	neutropenia	neutropenia	PPE/neutropenia	fatigue/fatigue
PFS (weeks)	17	16	12.1 (18)	9.1 versus 13.6	12.4 versus 14.4
OS (weeks)		15 (months)	47 (NA)	35.6 versus 41.3	50.8 versus 54

PPE: palmar-plantar erythrodysesthesia.

**Table 5:** Study result for platinum resistant recurrent ovarian cancer (combination).

	GEM/weekly PTX	GEM/PLD	PLD/Topotecan
Dose (mg/m^2^)	1000/80	1000/30	30/1
	day 1.8. 15/4w	day 1.8/day l/3w	day l/day l–5/3w

Author	Garcia et al. [12]	Ferrandina et al. [19]	Verhaar-Langereis et al. [20]
			
Number of patients	35	66	27
			
Response rate			
CR + PR (%)	40	21.6	28
			
+ SD (%)	77	53.9	72
			
PFS (months)	5.7	20 weeks	30 weeks
			
OS (months)	13.1	50 weeks	41 weeks

**Table 6:** Recommendation for secondary cytoreduction based on disease free interval and number of recurrence site.

Disease free	Single site	Multiple site	Carcinomatosis
interval		no carcinomatosis	
6–12 Mo	offer SC	consider SC	No SC
12–30 Mo	offer SC	offer SC	consider SC
>30 Mo	offer SC	offer SC	offer SC

DFI: disease free interval. SC: secondary cytoreduction. Chi et al. [21].

## References

[1] Herzog TJ (2004). Recurrent ovarian cancer: how important is it to treat to disease progression?. *Clinical Cancer Research*.

[2] Rose PG, Fusco N, Fluellen L, Rodriguez M (1998). Second-line therapy with paclitaxel and carboplatin for recurrent disease following first-line therapy with paclitaxel and platinum in ovarian or peritoneal carcinoma. *Journal of Clinical Oncology*.

[3] du Bois A, Lück HJ, Pfisterer J (2001). Second-line carboplatin and gemcitabine in platinum sensitive ovarian cancer—a dose-finding study by the Arbeitsgemeinschaft Gynäkologische Onkologie (AGO) Ovarian Cancer Study Group. *Annals of Oncology*.

[4] Power P, Stuart G, Oza A (2009). Efficacy of pegylated liposomal doxorubicin (PLD) plus carboplatin in ovarian cancer patients who recur within six to twelve months: a phase II study. *Gynecologic Oncology*.

[5] Ushijima K, Hirakawa T, Saitoh T Phase II study of docetaxel and carboplatin in patients with recurrent epithelial ovarian cancer after first-line treatment with paclitaxel and/or platinum treatment.

[6] The ICON and AGO Collaborators (2003). Paclitaxel plus platinum-based chemotherapy versus conventional platinum—based chemotherapy in women with relapsed ovarian cancer: the ICON4/AGO-OVAR-2.2 trial. *The Lancet*.

[7] González-Martín AJ, Calvo E, Bover I (2005). Randomized phase II trial of carboplatin versus paclitaxel and carboplatin in platinum-sensitive recurrent advanced ovarian carcinoma: a GEICO (Grupo Espa ñol de Investigación en Cáncer de Ovario) study. *Annals of Oncology*.

[8] Pfisterer J, Plante M, Vergote I (2006). Gemcitabine plus carboplatin compared with carboplatin in patients with platinum-sensitive recurrent ovarian cancer: an intergroup trial of the AGO-OVAR, the NCIC CTG, and the EORTC GCG. *Journal of Clinical Oncology*.

[9] Alberts DS, Liu PY, Wilczynski SP (2008). Randomized trial of pegylated liposomal doxorubicin (PLD) plus carboplatin versus carboplatin in platinum-sensitive (PS) patients with recurrent epithelial ovarian or peritoneal carcinoma after failure of initial platinum-based chemotherapy (Southwest Oncology Group Protocol S0200). *Gynecologic Oncology*.

[10] Pujiade-Lauraine E, Mahner S, Kaern J A randomized, phase III study of carboplatin and pegylated liposomal doxorubicin versus carboplatin and paclitaxel in relapsed platinum-sensitive ovarian cancer (OC): CALYPSO study of the Gynecologic Cancer Inter Group (GCIG).

[11] Markman M, Webster K, Zanotti K, Kulp B, Peterson G, Belinson J (2003). Phase 2 trial of single-agent gemcitabine in platinum-paclitaxel refractory ovarian cancer. *Gynecologic Oncology*.

[12] Garcia AA, O'Meara A, Bahador A (2004). Phase II study of gemcitabine and weekly paclitaxel in recurrent platinum-resistant ovarian cancer. *Gynecologic Oncology*.

[13] O'Malley DM, Azodi M, Makkenchery A (2005). Weekly topotecan in heavily pretreated patients with recurrent epithelial ovarian carcinoma. *Gynecologic Oncology*.

[14] Mutch DG, Orlando M, Goss T (2007). Randomized phase III trial of gemcitabine compared with pegylated liposomal doxorubicin in patients with platinum-resistant ovarian cancer. *Journal of Clinical Oncology*.

[15] Bookman MA, Malmström H, Bolis G (1998). Topotecan for the treatment of advanced epithelial ovarian cancer: an open-label phase II study in patients treated after prior chemotherapy that contained cisplatin or carboplatin and paclitaxel. *Journal of Clinical Oncology*.

[16] Gadducci A, Fuso L, Cosio S (2009). Are surveillance procedures of clinical benefit for patients treated for ovarian cancer? A retrospective Italian multicentric study. *International Journal of Gynecological Cancer*.

[17] Gadducci A, Sartori E, Maggino T (1997). Analysis of failures in patients with stage I ovarian cancer: an Italian multicenter study. *International Journal of Gynecological Cancer*.

[18] Hoskins P, Tu D, James K, Pater J, Koski B (1998). Factors predictive of survival after first relapse or progression in advanced epithelial ovarian carcinoma: a prediction tree analysis-derived model with test and validation groups. *Gynecologic Oncology*.

[19] Ferrandina G, Paris I, Ludovisi M (2005). Gemcitabine and liposomal doxorubicin in the salvage treatment of ovarian cancer: updated results and long-term survival. *Gynecologic Oncology*.

[20] Verhaar-Langereis M, Karakus A, Van Eijkeren M, Voest E, Witteveen E (2006). Phase II study of the combination of pegylated liposomal doxorubicin and topotecan in platinum-resistant ovarian cancer. *International Journal of Gynecological Cancer*.

[21] Chi DS, McCaughty K, Diaz JP (2006). Guidelines and selection criteria for secondary cytoreductive surgery in patients with recurrent, platinum-sensitive epithelial ovarian carcinoma. *Cancer*.

[22] Markman M, Rothman R, Hakes T (1991). Second-line platinum therapy in patients with ovarian cancer previously treated with cisplatin. *Journal of Clinical Oncology*.

[23] Gore ME, Fryatt I, Wiltshaw E, Dawson T (1990). Treatment of relapsed carcinoma of the ovary with cisplatin or carboplatin following initial treatment with these compounds. *Gynecologic Oncology*.

[24] Ozols RF (2002). Recurrent ovarian cancer: evidence-based treatment. *Journal of Clinical Oncology*.

[25] Thigpen JT, Blessing JA, Ball H, Hummel SJ, Barrett RJ (1994). Phase II trial of paclitaxel in patients with progressive ovarian carcinoma after platinum-based chemotherapy: a Gynecologic Oncology Group study. *Journal of Clinical Oncology*.

[26] Colombo N, Gore M (2007). Treatment of recurrent ovarian cancer relapsing 6–12 months post platinum-based chemotherapy. *Critical Reviews in Oncology/Hematology*.

[27] Spriggs D (2003). Optimal sequencing in the treatment of recurrent ovarian cancer. *Gynecologic Oncology*.

[28] Vasey PA, Jayson GC, Gordon A (2004). Phase III randomized trial of docetaxel-carboplatin versus paclitaxel-carboplatin as first-line chemotherpy for ovarian carcinoma. *Journal of the National Cancer Institute*.

[29] Ozols RF (2005). Treatment goals in ovarian cancer. *International Journal of Gynecological Cancer*.

[30] Muggia FM, Hainsworth JD, Jeffers S (1997). Phase II study of liposomal doxorubicin in refractory ovarian cancer: antitumor activity and toxicity modification by liposomal encapsulation. *Journal of Clinical Oncology*.

[31] Gordon AN, Granai CO, Rose PG (2000). Phase II study of liposomal doxorubicin in platinum- and paclitaxel-refractory epithelial ovarian cancer. *Journal of Clinical Oncology*.

[32] Rose PG (2005). Pegylated liposomal doxorubicin: optimizing the dosing schedule in ovarian cancer. *The Oncologist*.

[33] Thigpen JT, Aghajanian CA, Alberts DS (2005). Role of pegylated liposomal doxorubicin in ovarian cancer. *Gynecologic Oncology*.

[34] Sehouli J (2005). Review of gemcitabine-based combinations for platinum-resistant ovarian cancer. *International Journal of Gynecological Cancer*.

[35] Kudelka AP, Tresukosol D, Edwards CL (1996). Phase II study of intravenous topotecan as a 5-day infusion for refractory epithelial ovarian carcinoma. *Journal of Clinical Oncology*.

[36] Rodriguez M, Rose PG (2001). Improved therapeutic index of lower dose topotecan chemotherapy in recurrent ovarian cancer. *Gynecologic Oncology*.

[37] Markman M, Kennedy A, Webster K, Kulp B, Peterson G, Belinson J (2000). Phase 2 evaluation of topotecan administered on a 3-day schedule in the treatment of platinum- and paclitaxel-refractory ovarian cancer. *Gynecologic Oncology*.

[38] Gordon AN, Fleagle JT, Guthrie D, Parkin DE, Gore ME, Lacave AJ (2001). Recurrent epithelial ovarian carcinoma: a randomized phase III study of pegylated liposomal doxorubicin versus topotecan. *Journal of Clinical Oncology*.

[39] Tay E-H, Grant PT, Gebski V, Hacker NF (2002). Secondary cytoreductive surgery for recurrent epithelial ovarian cancer. *Obstetrics and Gynecology*.

[40] Eisenkop SM, Friedman RL, Spirtos NM (2000). The role of secondary cytoreductive surgery in the treatment of patients with recurrent epithelial ovarian carcinoma. *Cancer*.

[41] Gadducci A, Iacconi P, Cosio S, Fanucchi A, Cristofani R, Genazzani AR (2000). Complete salvage surgical cytoreduction improves further survival of patients with late recurrent ovarian cancer. *Gynecologic Oncology*.

[42] Onda T, Yoshikawa H, Yasugi T, Yamada M, Matsumoto K, Taketani Y (2005). Secondary cytoreductive surgery for recurrent epithelial ovarian carcinoma: proposal for patients selection. *British Journal of Cancer*.

